# In silico screening and heterologous expression of soluble dimethyl sulfide monooxygenases of microbial origin in *Escherichia coli*

**DOI:** 10.1007/s00253-022-12008-8

**Published:** 2022-06-17

**Authors:** Prasanth Karaiyan, Catherine Ching Han Chang, Eng-Seng Chan, Beng Ti Tey, Ramakrishnan Nagasundara Ramanan, Chien Wei Ooi

**Affiliations:** 1 Chemical Engineering Discipline, School of Engineering, Monash University Malaysia, Jalan Lagoon Selatan, 47500 Bandar Sunway, Selangor Malaysia; 2 Arkema Thiochemicals Sdn. Bhd., Jalan PJU 1A/7A OASIS Ara Damansara, 47301 Petaling Jaya, Selangor Darul Ehsan Malaysia; 3 Advanced Engineering Platform, Monash University Malaysia, Jalan Lagoon Selatan, 47500 Bandar Sunway, Selangor Malaysia

**Keywords:** Biocatalysts, DMS monooxygenase, *Escherichia coli*, In silico screening, Oxidoreductase activity, Recombinant expression

## Abstract

**Abstract:**

Sequence-based screening has been widely applied in the discovery of novel microbial enzymes. However, majority of the sequences in the genomic databases were annotated using computational approaches and lacks experimental characterization. Hence, the success in obtaining the functional biocatalysts with improved characteristics requires an efficient screening method that considers a wide array of factors. Recombinant expression of microbial enzymes is often hampered by the undesirable formation of inclusion body. Here, we present a systematic in silico screening method to identify the proteins expressible in soluble form and with the desired biological properties. The screening approach was adopted in the recombinant expression of dimethyl sulfide (DMS) monooxygenase in *Escherichia coli*. DMS monooxygenase, a two-component enzyme consisting of DmoA and DmoB subunits, was used as a model protein. The success rate of producing soluble and active DmoA is 71% (5 out of 7 genes). Interestingly, the soluble recombinant DmoA enzymes exhibited the NADH:FMN oxidoreductase activity in the absence of DmoB (second subunit), and the cofactor FMN, suggesting that DmoA is also an oxidoreductase. DmoA originated from *Janthinobacterium* sp. AD80 showed the maximum NADH oxidation activity (maximum reaction rate: 6.6 µM/min; specific activity: 133 µM/min/mg). This novel finding may allow DmoA to be used as an oxidoreductase biocatalyst for various industrial applications. The in silico gene screening methodology established from this study can increase the success rate of producing soluble and functional enzymes while avoiding the laborious trial and error involved in the screening of a large pool of genes available.

**Key points:**

• *A systematic gene screening method was demonstrated.*

• *DmoA is also an oxidoreductase capable of oxidizing NADH and reducing FMN.*

• *DmoA oxidizes NADH in the absence of external FMN.*

**Supplementary Information:**

The online version contains supplementary material available at 10.1007/s00253-022-12008-8.

## Introduction

Chemical catalysis is often claimed to be environmentally unfriendly due to the harsh operating conditions, generation of unwanted byproducts, and high cost of downstream processing (Lin and Tao 2017). Hence, biocatalysts are widely used in various industries as an alternative to chemical catalysts (Sheldon and Woodley 2018) because of their high selectivity, specificity, reusability, and non-toxicity (Bilal and Iqbal 2019; Sheldon and Brady 2019). Novel or improved biocatalysts can be discovered from natural microbial source or by database mining. Microbial screening approach is effective in uncovering new strains and species, but 99% of the identified microorganisms are unculturable or the culture of these microorganisms suffer from the low yield of the desired products (Lorenz and Eck 2004; van Rossum et al. 2013; Yun and Ryu 2005). However, some enzymes from harsh environments may exhibit remarkable catalytic properties and are worth of exploration. To utilize the biocatalysts from extremophiles, research groups have started mining the genomes of these unculturable microorganisms from gene database (Kelly et al. 2020). These proteins can be recombinantly expressed in tractable organisms such as *Escherichia coli* (*E. coli*) by introducing the enzyme-encoding gene into the host (Arnold 2001).

Sequence-based screening from gene database is one of the most cost-effective and time-saving methods in enzyme screening. The number of sequences in the gene database increased at the rate of 2%/month, and 50% of the deposited sequences were uncharacterized and hypothetical proteins with incorrect function annotations (Gerlt 2016). Therefore, the main challenge is to screen for the desired proteins from a large number of sequences deposited in the gene database. Homology search and evolutionary analysis are the most widely used strategies for screening the target proteins (Pearson 2013; Rebehmed et al. 2013). Sequence similarity search using basic local alignment search tool (BLAST) and phylogenetic analysis identifies homologous proteins that exhibit close phylogeny with the wild-type proteins (Kelly et al. 2020); however, screening based on similarity may miss some of the useful distantly related enzymes with better catalytic characteristics. Apart from these methods, key motifs and structural analysis were employed in the discovery of novel proteins (Höhne et al. 2010; Kelly et al. 2020; Mascotti et al. 2013). However, some putative proteins selected using the screening methods as stated above or extracted from the database were recombinantly expressed as inclusion bodies (IBs) in *E. coli* (Maruthamuthu and van Elsas 2017; Pire et al. 2001; Slomka et al. 2017). IBs are typically insoluble and biologically inactive (Chang et al. 2015). The expression of proteins in insoluble form is also undesirable, even though they could be refolded in vitro to attain catalytically active state. Furthermore, the recovery of soluble protein from IB fraction is low and time consuming due to multiple steps of washing, solubilization, and refolding (Chang et al. 2016). The protein solubility can be enhanced by (i) selecting a suitable promoter; (ii) co-expressing molecular and chemical chaperones; (iii) adding a fusion or small peptide tag at the N- or C-terminal end; (iv) changing the host strain; (v) optimizing the protein induction temperature, inducer concentration, and growth media; and (vi) adding osmolytes such as glucose, ethanol, glycerol, or sorbitol in the culture media (Abady et al. 2021; Duzenli and Okay 2020; Han et al. 2020; Mahmoud et al. 2021; Mamipour et al. 2017).

Bioinformatics tools predict the gene expression and soluble protein production by considering information including the codon usage, transcription, and translation efficiency. Gene expression is often hampered by the presence of rare codons. To improve the gene expression in *E. coli*, many algorithms of codon optimization convert the rare codons of heterologous genes to the most frequently used codons (Gould et al. 2014). The process of translation is regulated by factors such as messenger RNA (mRNA) secondary structures at translation initiation region (TIR), mRNA secondary structures of coding sequence, and minimum free energy (MFE) (Yin et al. 2016). Strong mRNA structures at the TIR were prohibitive to the protein production in prokaryotic systems (Gustafsson et al. 2012). Thus, the predictions of mRNA secondary structures and MFE by computational methods are a key to the successful expression of heterologous proteins. Apart from bioinformatic tools involving the information at gene level, numerous machine learning-based methods have been developed to predict the solubility of protein, based on its amino acid sequence (Agostini et al. 2014; Habibi et al. 2014; Hebditch et al. 2017; Hon et al. 2021; Khurana et al. 2018; Madani et al. 2021a; Magnan et al. 2009; Rawi et al. 2018; Smialowski et al. 2012; Sormanni et al. 2015). The solubility prediction tools can discriminate between soluble and insoluble proteins, which can avoid the trial-and-error approach of protein expression in *E. coli* (Chang et al. 2014). Even though there are many bioinformatic tools, there is no study evaluating the combination of bioinformatic tools in rendering a good outcome of soluble enzymes with desired characteristics.

The present work aims to establish a systematic in silico screening method for the screening of potential dimethyl sulfide (DMS) monooxygenases (EC 1.14.13.131) from a large pool of genes including the uncharacterized hypothetical sequences deposited in the NCBI database, and to recombinantly express the screened genes in soluble form. DMS monooxygenase, a sulfur dissimilatory enzyme, degrades DMS into methanethiol and formaldehyde (Boden et al. 2011; Boden and Hutt 2018; De Bont et al. 1981). Thus, DMS monooxygenase is a promising biocatalyst used in the bioremediation of sulfur pollutant; for example, dibenzothiophene (DBT) monooxygenase and DBT-sulfone monooxygenase are capable of desulfurizing DBT, an organosulfur pollutant generated from the combustion of fossil fuel (Gray et al. 2003; Su et al. 2018). DMS monooxygenase consists of two subunits: DmoA, a monooxygenase, and DmoB, an oxidoreductase. DmoB reduces flavin mononucleotide (FMN) to FMNH_2_ by oxidation of nicotinamide adenine dinucleotide (NADH), and subsequently, it transfers FMNH_2_ to the monooxygenase subunit, DmoA (Boden et al. 2011; Cao et al. 2018; Hammers et al. 2020). DmoA degrades DMS using molecular dioxygen (O_2_) and FMNH_2_ supplied by DmoB (Boden et al. 2011). To address the challenge of screening soluble enzymes from millions of uncharacterized sequences, we have developed a systematic in silico screening method. The in silico method is a combination of enzyme screening and optimization of the factors involved in the gene expression. Here, we report for the first time, the soluble recombinant expression and characterization of oxidoreductase activities of seven DmoA screened through in silico method. The recombinant DmoA enzymes were assayed for NADH oxidation activity both in the presence and absence of FMN. The DmoB enzymes were coupled with DmoA for analyzing the DMS degradation activity.

## Materials and methods

### Materials

BL21 Star (DE3) *E. coli* chemically competent cells, kanamycin, and FMN were purchased from Thermo Fisher Scientific, Waltham, USA. Luria Bertani broth and autoinduction media were purchased from Merck, Kenilworth, USA, and Formedium, Hunstanton, UK, respectively. Cell lysis buffer (FastBreak ™) was purchased from Promega, Madison, USA. NADH and FMN were purchased from Alfa Aesar, Tewksbury, USA. Protein assay kits, namely Bicinchoninic acid (BCA) and Bradford reagents, were acquired from Nacalai Tesque, Kyoto, Japan, and Bio-Rad, Hercules, USA, respectively. Protein purification kit nickel-nitrilotriacetic acid (Ni^2+^-NTA) resin column was purchased from QIAGEN, Hilden, Germany. DMS was purchased from Sigma-Aldrich, St. Louis, USA. The protein marker (Color Prestained Protein Standard, Broad Range, 11–245 kDa) was purchased from NEB Biolabs, Ipswich, USA. All other chemicals were obtained from Sigma-Aldrich, St. Louis, USA, unless otherwise stated.

### In silico* gene screening of potential DMS monooxygenases*

Firstly, sequences of nucleotide and amino acids encoding DMS monooxygenases were retrieved from the National Center for Biotechnology Information (NCBI) database with protein name “dimethylsulfide monooxygenase” and gene “*dmoA*.” Cluster database at high identity with tolerance (CD-HIT), a protein clustering algorithm, was used to eliminate protein sequences with 100% sequence identity. By using a multiple sequence alignment algorithm (ClustalW), the remaining protein sequences in the dataset was subjected to further classification into either homologous or non-homologous category based on 50% sequence identity threshold.

**Fig. 1:**
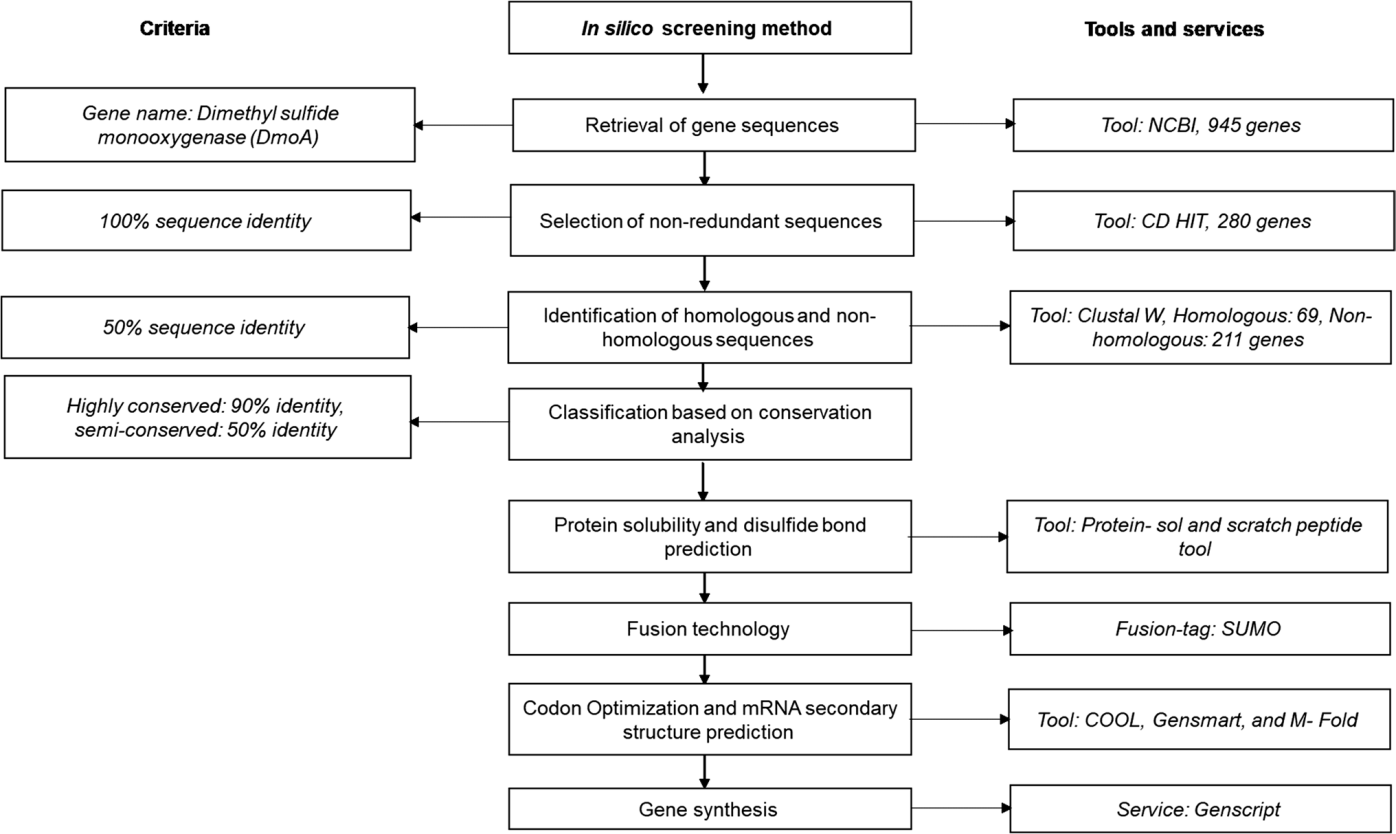
Overview of in silico gene screening. The methodology includes both screening of potential DmoA from NCBI database and improvement of recombinant expression of DmoA in *E. coli*

DMS monooxygenase from *Hyphomicrobium* (GenBank accession number: GQ980036) was compared with the homologous sequence (long-chain alkane monooxygenase (LadA), protein data bank (PDB) code: (3B9O)) to identify the conserved residues (active and binding site) residues. Based on the conserved residues involved in the binding of substrate and cofactors, all the putative sequences were manually aligned with DmoA1 and further classified into the categories of “highly conserved,” “semi-conserved,” and “non-conserved” based on 90%, 50%, and < 20% identity with the query sequence, respectively (Fig. S3). An overview of the in silico gene screening approach used in this study is given in Fig. 1.

### Bioinformatic tools for prediction of soluble heterologous protein expression

To improve the solubility of selected enzymes, the following bioinformatic tools were used in this study.

Protein-Sol, a sequence-based prediction tool, calculates the solubility score based on the amino acid sequence (Hebditch et al. 2017). Protein-Sol was run from https://protein-sol.manchester.ac.uk/software. Protein-Sol classifies proteins as either highly soluble or less soluble based on scaled solubility score. There is no threshold at which soluble and insoluble proteins are discriminated. For example, proteins with a scaled solubility score greater than 0.45 are highly soluble and scores less than 0.45 are predicted to be less soluble. For proteins predicted as less soluble, small ubiquitin-like modifier (SUMO) and glutathione S-transferase (GST) tags were added to the N-terminal end and the scores were recalculated.

DIpro (SCRATCH-protein predictor), a cysteine disulfide bond predictor, was used to predict the presence of disulfide bonds in protein and the number of disulfide bonds. The disulfide-bonded proteins will be targeted to the oxidizing periplasm and the proteins without disulfide bonds to the reducing cytoplasm. DIpro was accessible via the website http://scratch.proteomics.ics.uci.edu.

Codon optimization online (COOL) and Gensmart are the codon optimization tools that convert the rare codons in *dmoA* genes to the most frequently used codons. The gene sequences of codon-optimized *dmoA* were subjected to evaluation based on mRNA secondary structures at the TIR and the coding sequence of first 40 base pairs (bp). COOL customizes various codon optimization parameters such as codon adaptation index, codon pairing, individual codon usage, hidden stop codons, and mRNA secondary structure prediction, to achieve the optimal recombinant gene expression. Gensmart, a codon optimization tool offered by Genscript, has additional advantages when compared to COOL (Chin et al. 2014). Gensmart optimizes several parameters critical to the transcription (GC content, repeat sequences, cis-acting elements) and translation (mRNA secondary structures and internal chi sites). COOL was run from http://bioinfo.bti.a-star.edu.sg/COOL/. Gensmart optimization tool was accessed using Genscript services.

Vienna RNA package and M Fold predict mRNA secondary structures and MFE from both COOL- and Gensmart-optimized sequences. Vienna RNA Fold was run from http://rna.tbi.univie.ac.at/cgi-bin/RNAWebSuite/RNAfold.cgi and M fold was accessed from http://www.bioinfo.rpi.edu/applications/mfold/.

### Cloning, expression, and purification of DMS monooxygenases

Both *dmoA* and *dmoB* genes were synthesized using Genscript services and were cloned into pET-28b(+) vector between 5′-*Nco*I and 3′-*Xho*I restriction sites with a C-terminal polyhistidine tag (Genscript). The gene constructs were confirmed by DNA sequencing (Bio Basic). All the *dmoA*- or *dmoB*-containing plasmids were transformed into BL21 Star (DE3) chemically competent *E. coli* cells by heat shock method (Froger and Hall 2007; Ramalakshmi et al. 2021). A single colony was streaked and inoculated for primary culture at 37 °C in Luria Bertani broth. The primary culture (1%) was used as inoculum for secondary culture (Ramanan et al. 2010). The cells were grown at 37 °C in auto-induction media in the presence of 25 mg/mL of kanamycin. After reaching OD_600_ = 0.8, the culture was incubated at 18 °C (Infors HT Ecotron, Kuala Lumpur, Malaysia) and 0.7012 × *g* for 16 h. Next, the cells were harvested by centrifugation at 3944 × *g* for 6 min (Mierendorf et al. 2000). After suspending the pellet with 1× FastBreak ™ lysis buffer at a ratio of 1:3 (pellet:buffer), the suspension was centrifuged at 3944 × *g* for 20 min to collect the soluble fractions. The IB fraction was dissolved in an extraction buffer (50 mM Tris, 50 mM NaCl, 10 mM β-mercaptoethanol, 8 M urea, pH 8) under stirring for 20 min. The supernatant was then collected after the centrifugation of the mixture at 3944 × *g* for 45 min (Singh et al. 2015; Yang et al. 2011). The protein samples were then analyzed by sodium dodecyl sulfate polyacrylamide gel electrophoresis (SDS-PAGE) (Laemmli 1970).

The soluble supernatant was loaded onto a Ni^2+^-NTA resin column pre-equilibrated with a buffer (pH 8) made of 50 mM Tris HCl, 100 mM NaCl, and 10 mM imidazole. After flushing the column with a wash buffer (50 mM Tris HCl, 100 mM NaCl, 20 mM imidazole, pH 8), the proteins were eluted from the column with an elution buffer (50 mM Tris HCl, 100 mM NaCl, 200 mM imidazole, pH 8) (Cao et al. 2018). The eluted fractions, along with all the fractions obtained from the washing and flow-through steps, were analyzed using SDS-PAGE.

### Protein quantification

The total soluble protein content was quantified using the BCA kit (Nacalai Tesque) according to the instruction from the manufacturer (Smith et al. 1985). Total insoluble protein was measured using Bradford method (Bradford 1976). Calibration curves were prepared from the standard solutions of bovine serum albumin. Recombinant proteins were analyzed using SDS-PAGE (Smith 1984), whereas soluble and insoluble protein were quantified based on the band intensities (Image Lab) in SDS-PAGE gel.

### NADH oxidation and NADH:FMN oxidoreduction assay

NADH oxidation activities of both soluble DmoA and DmoB subunits were tested individually. The oxidation of NADH by DmoA and DmoB was monitored at 350 nm using a UV–Visible spectrophotometer (Klingenberg 1974). The assay was carried out in 96-well microplates at 30 °C. The reaction mixture (300 µL) was composed of 1× PBS buffer pH 7.4, 500 µM NADH, and 10 µM enzyme. The reaction mixture without enzyme and soluble fractions from *E. coli* BL21 star (DE3) cells containing empty vector [pET-28b(+)] was used as a negative control. NADH oxidation was monitored by measuring the decrease in absorbance, and the concentration was calculated with the extension coefficient of NADH (6.22 mM^−1^ cm^−1^).

NADH:FMN oxidoreductase assay was carried out under the above-mentioned conditions with the addition of 3 µM FMN in the reaction mixture.

### DMS monooxygenase assay

DMS monooxygenase activity was assayed by quantifying DMS (reactant) and methanethiol (product) in the headspace through gas chromatography (GC). The DMS degradation reaction was carried out in a headspace GC crimped vial at 30 °C shaking at 1.0956 × *g*. The reaction mixture (4 mL) comprises 20 mM piperazine-N, N′-bis (2-ethanesulfonic acid) (PIPES) buffer, pH 7.4, 1 mM NADH, 3 µM FMN, 5 µM dithiothreitol (DTT), 5 µM Mohr’s salt, and 2 mg/mL enzyme (DmoA subunit). The reaction was initiated by the addition of 1 mM DMS (Boden et al. 2011).

The volatile compounds were separated and analyzed by GS-GASPRO column (60 m × 320 µm) installed on GC system (Agilent 7890A) coupled with a flame ionization detector (FID) and a headspace autosampler (Agilent 7697A). The chromatography operation was programmed as follows: an initial oven temperature at 90 °C was held for 3 min and then increased to 200 °C at 30 °C/min and held for 17 min. The injection port was maintained at 150 °C. The compounds were detected using the FID maintained at 250 °C. The flow rates of air and hydrogen were set at 400 mL/min and 35 mL/min, respectively. The helium gas was injected at 3 mL/min and 82.389 kPa.

The temperatures of headspace oven, sample loop, and transfer line were maintained at 35 °C, 40 °C, and 50 °C, respectively. Before the sample injection, the vials were incubated at 30 °C for 15 min. The injection time was held at 0.5 min. The GC run time was 28 min. The peak areas from the chromatograms were taken for analysis.

## Results

### *Gene selection through *in silico* screening*

The established in silico gene screening methodology can be used to screen and characterize diverse panel of DMS monooxygenases. The following characteristics were taken into consideration for enzyme screening: (i) gene annotation, (ii) sequence redundancy and identity, (iii) active site residue analysis, (iv) isoelectric point (pI) value and solubility score of protein, (v) disulfide bond prediction, (vi) mRNA secondary structure prediction, (vii) microbial origin and toxicity. DMS monooxygenase from *Hyphomicrobium* sp. is the only well-characterized flavin-linked monooxygenase of the luciferase family. DMS degradation activity was also observed in *Arthrobacter* (Borodina et al. 2002) and *Thiobacillus* (Kanagawa and Kelly 1986), but no comprehensive study on the gene responsible for this activity was reported. The crystal structure of DmoA displayed triosephosphate isomerase (TIM) barrel made up of eight α-helices and eight β-strands (Cao et al. 2018). To date, the monooxygenase subunit (DmoA) from these bacterial strains has not been purified or characterized. Recently, a potential recombinant flavin reductase (DmoB) from *Arthrobacter globiformis* NBRC 1213 was characterized, while a putative DMS C-monooxygenase was predicted using phylogenetic analysis (Hammers et al. 2020). As of February 2019, a total of 945 putative sequences annotated as DMS monooxygenase were retrieved from the database. Among 945 sequences, only two enzymes were characterized: one gene was found to encode for alkanesulfonate monooxygenase, and the other gene from *Hyphomicrobium* sp. (Uniprot ID: E9JFX9.1, PDB code: 6ak1) encodes for DMS monooxygenase (Cao et al. 2018). The amino acid sequence from *Hyphomicrobium* sp. was used as a query sequence to screen the other novel genes annotated as DMS monooxygenase. After elimination of redundant sequences, the remaining 280 non-redundant sequences were subsequently analyzed through multiple sequence alignment (Fig. S1).

To identify the homologous proteins, multiple sequence alignment of the selected non-redundant sequences was performed. Classification using ClustalW yielded 69 homologous and 211 non-homologous proteins. A protein sequence (GenBank accession number: ODA67029.1) from the homologous sequence set gave the highest identity (77.77%). Sequence alignment of most of the proteins from non-homologous sequence set with *Hyphomicrobium* sp. showed > 25% identity except for a few sequences, namely Q845S8.1 (13.1%), RDI84207.1 (12.5%), RDI80964.1 (11%), RDI78335.1 (12.2%), ANC29693.1 (15.6%), and KZM35786.1 (16.4%). Finally, 91 DmoA-encoding proteins made from 27 homologous and 64 non-homologous sets were selected (Fig. S2).

Conserved residues play a vital role in the biological function or structural stability of proteins. The conserved binding residues were identified by sequence alignment of DMS monooxygenase (DmoA) with LadA, which shows 50% sequence identity and shares similar structure with DmoA (Cao et al. 2018). The binding residues of the DMS monooxygenase were found to be F10, M12, H17, Q18, ADV(57–59) Y63, Q79, S137, Y138, Y158, A227, ASG(229–231) F245, and GLG(366–368). These residues determine the substrate binding and catalytic activity. The substitution of these residues with different amino acids will have an impact on catalytic activity and substrate range. Thus, the selection of the proteins based on binding residues enables the identification of enzyme with improved activity. From the residue’s conservation analysis (Table S1), the amino acid residues F10, M12, H17, A227, F245, G366, and G368 are present in all the homologous sequences. Similarly, Y63, Y138, and Y158 were also noticed in all the sequences. The N-terminal amino acid Q18 is also present in almost all the sequences except for three sequences where the Q is replaced by L or Y. Only very few sequences in the non-homologous set were found to have the conserved amino acid residues throughout the sequences. Non-conserved proteins from both sets (homologous set: 1; non-homologous set: 10) were eliminated because the percentage of amino acid substitutions in their sequences is 90%.

Following the elimination of non-conversed proteins, the solubility scores of highly conserved and semi-conversed proteins from both homologous and non-homologous sets were predicted using Protein-Sol tool, and the highly soluble proteins were selected from each set. In addition to the predicted solubility score, the pI value of the protein and the microbial origin were taken into consideration when selecting the suitable protein candidates for this study. Based on the screening, most of the DMS monooxygenases were found to be originated from mesophilic organisms with a pI ranging from 4.7 to 7.2. Finally, seven proteins originated from different microorganisms (mesophilic, thermophilic, psychrophilic) and with different pI values (pI: 5 to 6.45) were selected (see Table 1). Extremophilic organisms such as psychrophiles and thermophiles are unculturable, and the wild mesophilic organisms require expensive media and give less yield. Therefore, we aimed to express these selected DmoA proteins (as originated from the extremophiles) heterologously in *E. coli*. Based on Protein-Sol prediction, four proteins (DmoA2, DmoA5, DmoA6, and DmoA7) were predicted to have a lower scaled solubility score. A commercial solubilization tag, SUMO tag, was added at the N-terminal end of these proteins to increase the protein solubility. SUMO and GST tags were added to the amino acid sequence of proteins that bear a lower scaled solubility; we found that the addition of a SUMO tag at the N-terminal end of the protein gives a higher solubility score than the addition of GST tag. Based on our study, the use of a SUMO tag at the N-terminal end was observed to give a higher scaled solubility score when compared to GST tag. Earlier study also reported that the expression of fusion tags such as GST, maltose-binding protein (MBP), and N-utilizing substance A protein (NusA) requires higher metabolic energy than SUMO (Waugh 2005). Even after addition of SUMO tag, the proteins were predicted to be less soluble. These four proteins with a lower scaled solubility score were also observed to have higher pI value at 6 and above, as compared to other proteins which were predicted to have a higher solubility score. The binding residues and gene details are shown in Table S1.

**Table 1:** DMS monooxygenases selected from in silico screening

Gene	GenBank accession number	Microbial origin	Organism characteristics	% Identity^A^	Protein solubility score^B^	Protein molecular weight (kDa)	pI
DmoA1	E9JFX9.1	*Hyphomicrobium* sp.	Mesophilic, methanotrophs, Gram negative	100	0.422	53.12	5.3
DmoA2	KIR16147.1	*Pseudomonas fluorescens*	Psychrophilic, Gram negative	50.84	0.35 (0.26)	52.66	6.1
DmoA3	AKP76571.1	*Bacillus megaterium* Q3	Mesophilic, Gram positive	51.29	0.459	52.26	5.7
DmoA4	ODA67029.1	*Methyloligella halotolerans*	Mesophilic, methanotrophs, Gram negative, halotolerant	77.77	0.542	46.43	5.0
DmoA5	PMQ09592.1	*Janthinobacterium* sp. AD80	Mesophilic, Gram negative	48.16	0.361 (0.25)	50.91	6.4
DmoA6	KXZ64626.1	*Acinetobacter venetianus*	Mesophilic, bioremediation, Gram negative	42.97	0.359 (0.25)	53.29	6.3
DmoA7	KPC99150.1	*Geobacillus* sp. BCO2	Thermophile, Gram positive	42.49	0.346 (0.30)	44.46	6.0

^A^Calculated by aligning the sequences with DMS monooxygenase from *Hyphomicrobium* sp.

^B^SUMO tag was added to proteins with low solubility (DmoA2, DmoA5, DmoA6, and DmoA7) and the solubility scores predicted without the addition of SUMO tag were provided in parentheses

Expression of recombinant proteins containing disulfide bonds in cytoplasm of *E. coli* results in the formation of IBs due to the absence of oxidative environment in cytoplasm. These disulfide-bonded proteins have to be targeted to the periplasm for soluble protein production (Chang et al. 2016). Therefore, it is necessary to predict if a protein has any disulfide bonds, by using DIpro tool. Based on DIpro prediction, all seven of the selected proteins were confirmed not containing any disulfide bonds, allowing them to be expressed in the cytoplasm of *E. coli*.

**Fig. 2:**
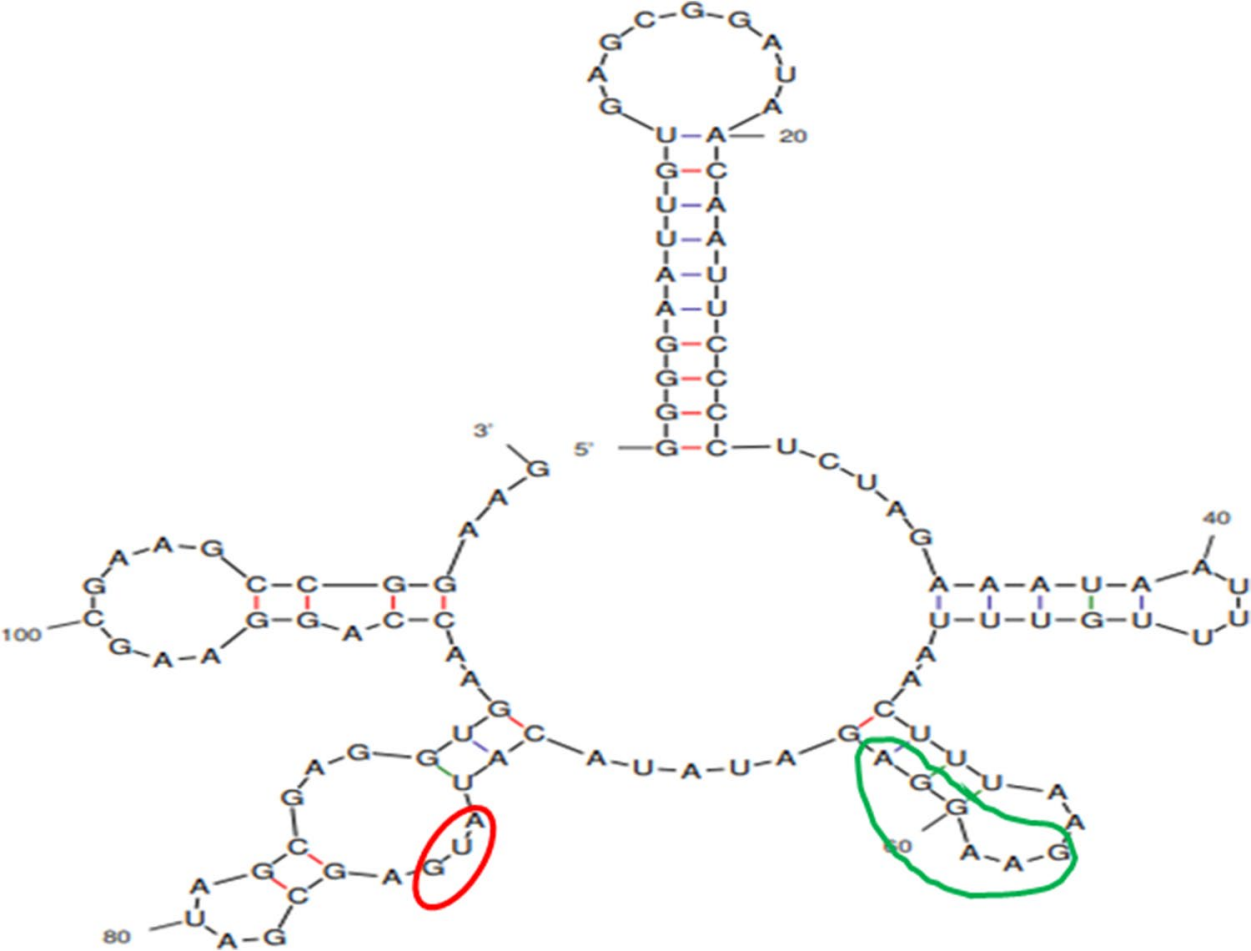
Diagram of predicted mRNA secondary structure expressed from TIR region in pET-28b(+) vector incorporated with SUMO tag. The red- and green-colored zones indicate start codon and Shine-Dalgarno sequence, respectively

The codon optimization strategy has been proven effective in improving the level of protein expression by increasing the rate of translation elongation (Mauro 2018). Therefore, the selected amino acid sequences were subjected to the analysis by COOL and Gensmart codon optimization tools for comparison studies. The sequences optimized using COOL and Gensmart were subsequently analyzed by Vienna RNA package and M Fold to predict the mRNA secondary structure and MFE. The formation of long stem-loop and intricate secondary structures in mRNA (hereinafter called “complex secondary structures”) at 5′ end, around ribosomal binding site (RBS), and start codon can block the ribosomes and thereby inhibiting the translation initiation (Yin et al. 2016). Hence, it is necessary to reduce the complex secondary structures of mRNA with increased MFE at the TIR. Many studies have shown that mRNA secondary structures at the TIR regulates the translation efficiency (Behloul et al. 2017; Gaspar et al. 2013; Hess et al. 2015; Roy et al. 2017; Yutaka et al. 2019). Thus, we analyzed the mRNA secondary structures and MFE values for the TIR portion with the first 40 base pairs of the gene sequence (Table S2). The structures with MFE value higher than − 6 kcal/mol do not decrease the translational efficiency (de Smit and van Duin 1994). Though the total MFE values for all the *dmoA* gene sequences are highly negative, the MFE values for RBS and start codon were higher than − 6 kcal/mol, hinting that the translational efficiency will not be severely affected. Most of the *dmoA* genes optimized by Gensmart were observed to form less complex secondary structures of mRNA at the TIR than that by the COOL-optimized sequences, except *dmoA4*. Hence, Gensmart-optimized gene sequences of *dmoA*1 (GenBank accession number: OM906818), *dmoA*2 (GenBank accession number: OM906819), *dmoA*3 (GenBank accession number: OM906820), *dmoA*5 (GenBank accession number: OM906822), *dmoA*6 (GenBank accession number: OM906823), and *dmoA*7 (GenBank accession number: OM906824), along with COOL-optimized gene sequence of *dmoA*4 (GenBank accession number: OM906821), were adopted in the subsequent protein expression studies. An example of predicted mRNA secondary structure expressed from TIR region at pET-28b(+) vector incorporated with SUMO tag is shown in Fig. 2.

### Recombinant expression of DMS monooxygenase in *E. coli*

**Fig. 3:**
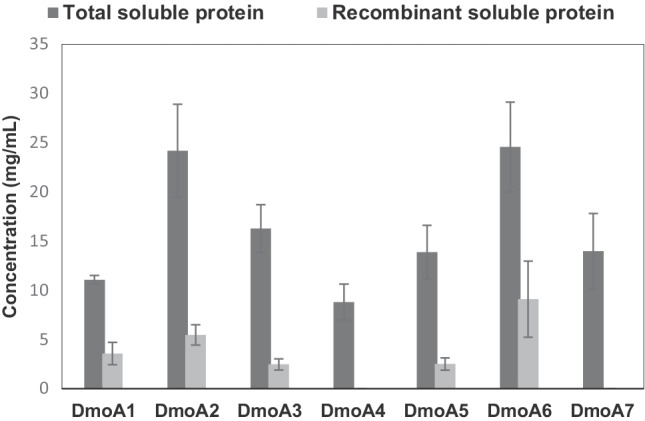
Production of DMS monooxygenases (DmoA) in *E. coli.* Data are shown as mean values from triplicates with corresponding standard error bars

DMS monooxygenases selected in accordance with in silico gene screening methodology were validated by in vivo studies. Hammers et al. (2020) reported the recombinant expression of DmoA from *Hyphomicrobium* sp. (DmoA1) and the *dmoA*1 gene was included in our study as a positive control. High soluble protein expression of DmoA2 (*Pseudomonas fluorescens*) and DmoA6 (*Acinetobacter venetianus*) was observed, with their concentrations at 5.4 mg/mL and 9.0 mg/mL, respectively (Fig. 3).

**Fig. 4:**
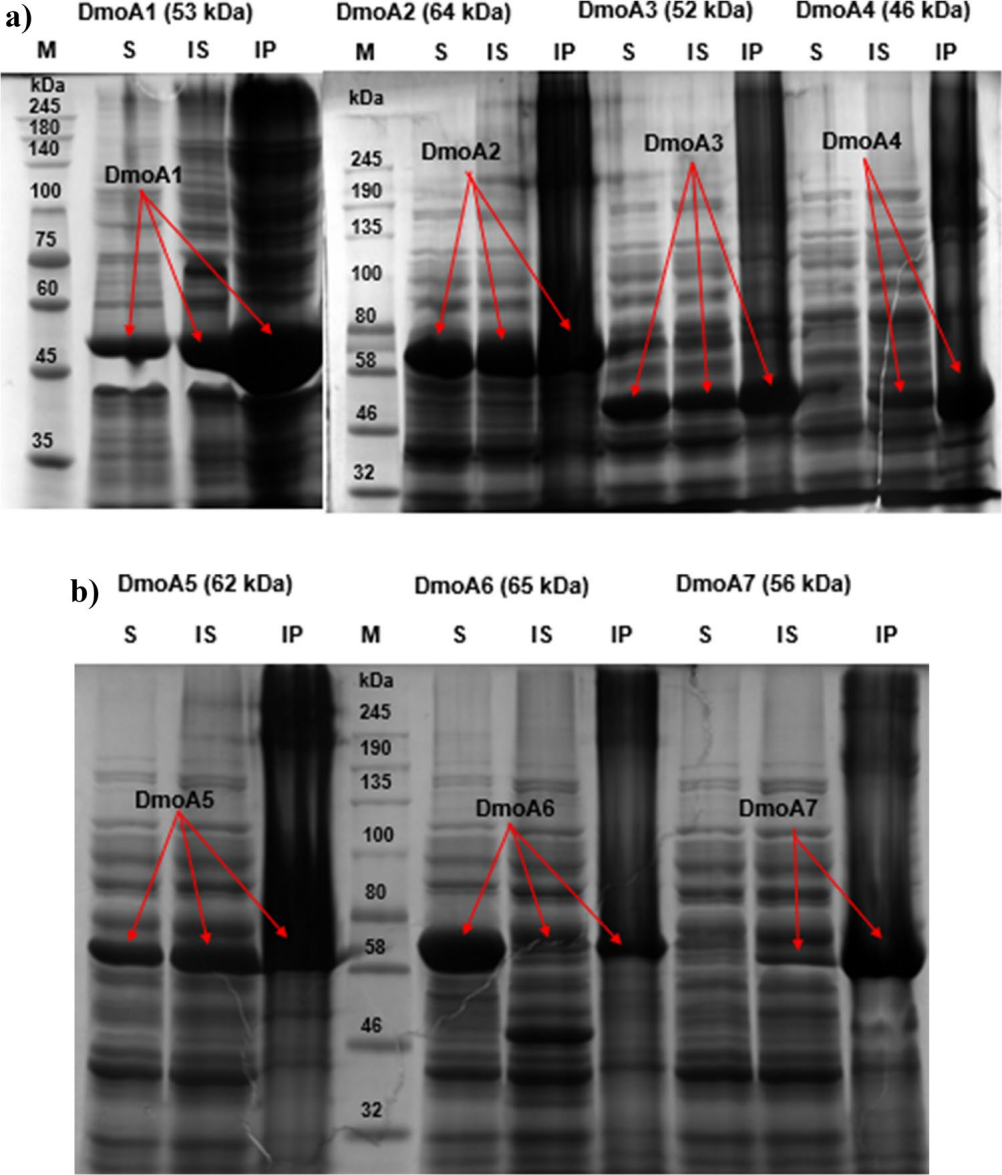
SDS-PAGE gels showing recombinant expression of DMS monooxygenases in *E. coli*. **a** DmoA1-DmoA4. **b** DmoA5-DmoA7. M, protein marker; S, soluble protein; IS, insoluble supernatant; IP, insoluble pellet

As shown in Fig. 4, seven recombinant strains expressing the *dmoA* genes resulted in the protein production, either as soluble or insoluble proteins. Expression of soluble protein from most of the gene constructs was observed, except DmoA4 and DmoA7. The DmoA2, DmoA5, DmoA6, and DmoA7 were predicted to have a lower probability of soluble expression even after addition of SUMO tag during in silico gene screening. However, addition of N-terminal SUMO tag resulted in 75% success rate of producing soluble proteins (3 out of 4), which shows that there are high chances of soluble expression for of the proteins with a solubility score greater than 0.3. Based on the solubility score generated from Protein-Sol tool, one false positive was observed. DmoA4 was predicted to be highly soluble based on its amino acid sequence, but it was produced as IBs.

### Expression and purification of recombinant flavin reductases (DmoB)

DMS monooxygenase was reported as a two-component enzyme, and both subunits are needed for the DMS degradation activity (Boden et al. 2011; Hammers et al. 2020). The gene encoding for DmoB from *Hyphomicrobium* sp. has not been characterized to date. Therefore, four DmoB were selected from the literature to study DMS degradation activity (Table 2) and the selected DmoB enzymes were codon-optimized by Gensmart tool to increase their success rate of recombinant expression. The codon-optimized *dmoB*1 (GenBank accession number: OM906825), *dmoB*2 (GenBank accession number: OM906826), *dmoB*3 (GenBank accession number: OM906827), and *dmoB*4 (GenBank accession number: OM906828) were recombinantly expressed in *E. coli*.

**Table 2:** Selected DMS monooxygenase’s second subunits (DmoB)

Code name	Gene	GenBank accession number	Microorganism	Protein molecular weight (kDa)	Reference
DmoB1	SsuE	CAB40389.1	*E. coli*	22	(Eichhorn et al. 1999)
DmoB2	Orf136	n/a	*Hyphomicrobium* sp.	17	(Boden et al. 2011)
DmoB3	Orf176	ADL39576.1	*Hyphomicrobium* sp.	20	(Boden et al. 2011)
DmoB4	Fre	AAA23806	*E. coli*	27	(Xun and Sandvik 2000)

**Fig. 5:**
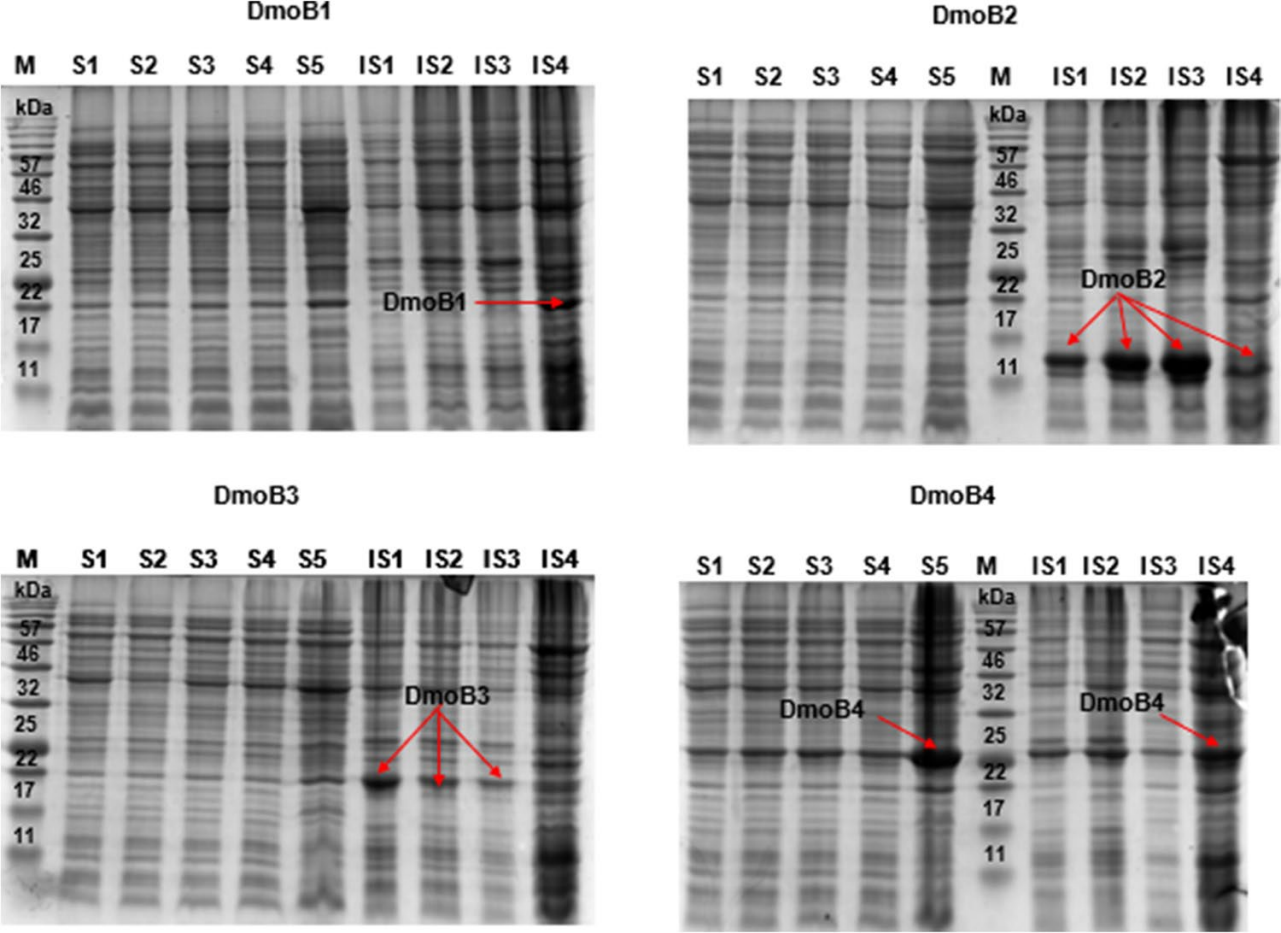
SDS-PAGE gels showing recombinant expression of DmoB1-DmoB4 in *E. coli*. M, protein marker; S1–S5, soluble fractions collected at 3, 4, 6, 7, and 14 h, respectively. IS1-IS4, insoluble fractions collected at 4, 6, 7, and 14 h, respectively

Protein samples obtained from the recombinant expression of all *dmoB* genes after 14 h were analyzed using SDS-PAGE and the results are shown in Fig. 5. Time-dependent protein profiling was done for all the DmoB enzymes to evaluate the expression level of soluble protein. Among the four recombinant flavin reductases included in this study, DmoB4 is the only protein expressed in soluble form (Fig. 5). Although expression of soluble protein was observed for the codon-optimized *dmoB1* (as shown by the bands in the SDS-PAGE gel), the subsequent His-tag purification of the fractions of soluble proteins confirmed their identity as host protein instead of the target recombinant DmoB1 containing His-tag (Fig. S4). The expression of both *dmoB2* (Orf136) and *dmoB4* (Orf 176) from *Hyphomicrobium* sp. resulted in the production of insoluble proteins. His-tag purified DmoB4 was identified as the protein band with an actual size of 27 kDa in the SDS-PAGE gel (Fig. S4). The actual molecular weight of DmoB2 is 17 kDa, but the His-tag purified DmoB2 was present as a faint band of 15 kDa in the lane of His-tag purified soluble fractions (Fig. S4); the recombinant expression of DmoB2 was confirmed, and the slight deviation in the molecular weight obtained could be due to peptide cleavage.

### DmoA as an oxidoreductase

**Fig. 6:**
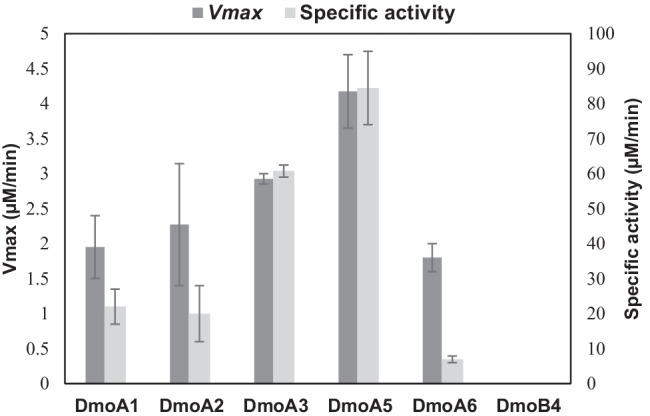
NADH oxidation activity of DmoA and DmoB in the absence of FMN. Data are shown as means of duplicates with the corresponding standard error bars

NADH oxidation activity was exhibited by the soluble DmoA enzymes in the absence of FMN. The highest NADH oxidation activity (*V*max) was exhibited by DmoA from *Janthinobacterium* sp. AD80 (DmoA5) (Fig. 6 and Fig. S6). DmoA6 from *Acinetobacter venetianus* showed a low activity of NADH oxidation. No NADH oxidation activity was observed in the soluble fractions collected from *E. coli* BL21 star (DE3) expressing empty vector.

**Fig. 7:**
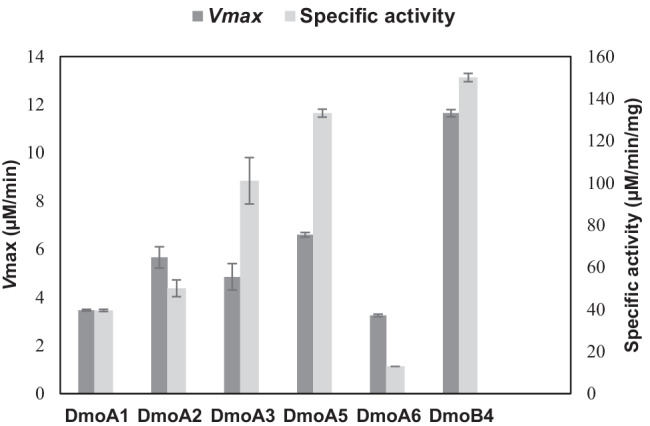
NADH:FMN activity of DmoA and DmoB. Data are shown as means of duplicates with the corresponding standard error bars

NADH oxidation and FMN reduction activities of the soluble DmoA enzymes in the absence of DmoB were first reported in this study, suggesting that the DMS monooxygenases included in this study could also be NADH:FMN oxidoreductase enzymes. In the presence of FMN, the NADH: FMN oxidoreduction activity of all soluble DmoA enzymes was observed to be higher than the NADH oxidation activity, indicating them as FMNH_2_-dependent monooxygenases (Fig. 7 and Fig. S7). The highest specific activity was exhibited by DmoA5 (133.15 µM/min/mg). However, the purified soluble DmoA enzymes did not show any NADH oxidation activity, which might be due to the loss of bound FMN during purification. Hence, restoration of activity was attempted by incubation on ice for 1 h with various concentrations of FMN (0.5, 3, 5, 10, and 20 µM), but no NADH oxidation activity was observed after the restoration attempt.

### Oxidoreductase activities of DmoB

NADH oxidation activities of DmoB2 and DmoB4 was tested in the presence or absence of 0.5 µM FMN. However, no NADH oxidation activity was observed in the absence of FMN (data not shown). Both DmoB2 and DmoB4 showed NADH oxidation activity in the presence of FMN. Among the two enzymes, DmoB4 showed the highest NADH:FMN oxidoreduction activity. Recombinant flavin reductase (DmoB4), which is originated from *E. coli* and highly soluble, exhibited NADH:FMN oxidation activity (Fig. S5). The *V*max and specific activity of His-tag purified DmoB4 were found to be 11.3 µM/min and 7229 µM/min/mg, respectively. When compared to the NADH:FMN oxidoreduction activities of DmoA, the soluble and His-tag purified fractions of DmoB4 showed a higher oxidoreduction activity. Interestingly, though DmoB2 from *Hyphomicrobium* sp. (Orf 136) was not expressed in soluble form, it showed NADH:FMN oxidoreduction activity in the presence of 3 µM FMN and no oxidoreduction activity was observed in the absence of FMN (data not shown); this clearly indicates that the enzyme does not contain any bound flavins. The specific activity of DmoB2 soluble fraction was 73 µM/min/mg, whereas the purified fractions did not show activity.

### DMS degradation activity of DmoA and DmoB (DmoAB)

The DMS (substrate) degradation activity of all soluble DmoA enzymes was tested using the soluble fractions and His-tag purified DmoA, with the addition of FMN and NADH to the reaction mixture. The reaction was conducted at different time intervals (Table S3). There was no formation of methanethiol detected from the biocatalytic reaction medium containing soluble and purified DmoA fractions. Although the soluble DmoA fractions exhibit oxidoreductase activity, the expected product (methanethiol) was not detected. The main drawback of purified enzymes is the loss of DmoA oxidoreductase activity after purification, resulted in the necessity to add a flavin reductase (DmoB) to supply FMNH_2_ for the DMS degradation activity. The production of methanethiol by the DMS monooxygenase from native *Hyphomicrobium* sp. and the DmoAB recombinantly expressed in *E. coli* occurred only when both the subunits (DmoAB) were present in equimolar concentration (Boden et al. 2011; Hammers et al. 2020). The combination of soluble DmoA and DmoB (i.e., DmoB2 or DmoB4) in equimolar concentration did not result in methanethiol production. These results strongly suggest that the investigated recombinant DMS monooxygenase is capable of depleting only NADH and not DMS in the absence of a suitable second subunit. The future works shall focus on the exploration of DmoB that could be coupled with the screened soluble DmoA for evaluation of the DMS degradation activities.

## Discussion

In this study, a systematic in silico screening methodology was established for identification of DMS monooxygenase candidates with the needed enzymatic activity and that could be recombinantly expressed in soluble form by *E. coli*. The genomic databases screening for the desired enzymes could be challenging when there is a vast number of putative sequences to be screened; moreover, recombinant expression of foreign gene in *E. coli* often results in IB formation or cell death. Hence, our in silico screening method considered a number of key factors such as sequence homology, conversation analysis, and elimination of toxic and fusion proteins to improve the success rate of obtaining functional DmoA enzymes.

Our screening method suggested seven candidates of DmoA originating from different microorganisms. All the seven recombinant DmoA were successfully expressed in *E. coli* BL21 Star (DE3), which indicates that the codon optimization is an effective strategy to maximize the protein expression. Codon optimization improves the translation efficiency of foreign gene by converting the nucleotide sequence of one species to another (Fu et al. 2020). Among the screened seven DmoA enzymes, five were expressed in a soluble form. This shows that the protein solubility prediction tools can increase the success rate of soluble expression with minimal runs of wet-lab experiments. To improve the accuracy of protein solubility prediction, the newer tools such as SoluProt (Hon et al. 2021) and DSResSol (Madani et al. 2021b) may be adopted in the future studies. It was claimed that the prediction accuracy and performance of SoluProt exceed the currently available tools (Ghomi et al. 2020).

The success rate of obtaining soluble DmoA screened using our in silico gene screening method is 71% soluble proteins (5 out of 7 genes). The achievement is considered remarkable when compared to other reported screening approaches due to the incorporation of mRNA secondary structure prediction, disulfide bond prediction, fusion technology, and elimination of toxic microorganisms. For example, recombinant expression of putative esterases screened through metagenomic approach resulted in only 9% soluble expression (1 out of 11) (Terahara et al. 2010). In *E. coli* BL21 (DE3), the expression of eight novel glycosyl hydrolases retrieved from a metagenomic library resulted in one of eight proteins being present in soluble fractions (12.5% of success rate) (Maruthamuthu and van Elsas 2017). The expression of 20 putative haloalkane dehalogenases screened through EnzymeMiner provided 60% soluble expression (12 out of 20 genes) (Vanacek et al. 2018). EnzymeMiner is a web-based tool that was developed for the automated screening and selection of soluble haloalkane dehalogenases (Hon et al. 2020). To further improve the soluble protein yield, factors such as the appropriate expression vector, strength of the promoter, fermentation conditions, and expression strain must be considered (Packiam et al. 2020).

DmoA from *Hyphomicrobium* sp. is the only characterized enzyme (Boden et al. 2011; Hammers et al. 2020). Here, we report for the first time, the recombinant expression and biochemical characterization of six putative DmoA retrieved from the NCBI database. Based on the data from our study, the recombinantly expressed DmoA subunit encoded by a single gene can act as a NADH:FMN oxidoreductase. Interestingly, NADH oxidation activity of DmoA in the absence of FMN was observed, indicating the possibility of DmoA to be a covalent flavoprotein or flavin-containing monooxygenase. DmoA from mesophilic microorganisms, namely *Bacillus megaterium* Q3 and *Janthinobacterium* sp. AD80, showed the highest NADH oxidation and NADH:FMN oxidoreduction activities. DMS monooxygenase from the native *Hyphomicrobium* sp. was also shown to oxidize NADH in the absence of FMN (De Bont et al. 1981). This suggests that DMS monooxygenase is an external monooxygenase containing the tightly bound flavin (EC 1.14.13) and is capable of utilizing NADH as reducing power. External monooxygenases are categorized into six subclasses (A–F) based on the sequence and structure (Van Berkel et al. 2006). Most of the two-component monooxygenases do not contain a tightly bound or covalently bonded FMN as a prosthetic group, and they depend on flavin reductase to supply the reduced FMN (Ellis 2010). Flavin containing monooxygenases such as N-hydroxylating monooxygenases, cyclohexanone monooxygenases, and 4-hydroxybenzoate 3-monooxygenase belonging to classes A or B contain a tightly bound flavin group (Van Berkel et al. 2006). The ability of DmoA from *Hyphomicrobium* sp. and other four soluble putative DmoA (i.e., DmoA2, DmoA3, DmoA5, and DmoA6) to catalyze NADH:FMN oxidoreduction is an interesting finding from our study.

Based on the oxidoreductase activities of DmoA and DmoB, we postulate that the DmoB2 (Orf 136) does not contain the tightly bound flavin group and it belongs to the class II flavin reductases, whereas DmoA may belong to class I flavin reductases containing bound FMN. Flavin reductases associated with monooxygenases are classified into two classes. Class I enzymes contain bound flavin and are referred as standard flavoprotein, e.g., the sulfite reductase (Fre) from *E. coli* and Frp reductase from *Vibrio harveyi* (Coves et al. 1993; Lei et al. 1994); class II enzymes do not contain any bound flavin groups and are defined as non-standard flavoproteins, e.g., SsuE from *E. coli* (Gao et al. 2005).

The soluble fractions of DmoA subunits were found to catalyze the NADH:FMN oxidoreduction, but not the substrate DMS. Although DmoA1 (*Hyphomicrobium* sp.) and DmoB2 exhibited NADH:FMN oxidoreduction activity, the coupling of DmoB2 with DmoA1 did not yield methanethiol. This indicates the importance of coupling a potential second subunit (DmoB) with DmoA in expressing the DMS degradation activity. The flavin reductases can either form a stable or transient complex with its monooxygenase. A stable protein–protein interaction between alkanesulfonate monooxygenase (SsuD) and flavin reductase (SsuE) was observed (Dayal et al. 2015). On the other hand, a transient complex was observed between styrene monooxygenase (SMOA) and NADH-specific flavin reductase (SMOB) (Kantz et al. 2005). Protein–protein interaction was also observed in other two-component monooxygenases including EDTA monooxygenase (EmoA) (Jun et al. 2016), bacterial luciferase from *Vibrio harveyi* (Low and Tu 2003), and *p*-hydroxyphenylacetate monooxygenase (HpaA) from *Pseudomonas aeruginosa* (Chakraborty et al. 2010). The future works shall focus on the exploration of DmoB that could be coupled with the screened soluble DmoA for evaluation of the DMS degradation activities.

In conclusion, the robust enzyme screening method described in this study is able to screen the potential candidates that can be recombinantly expressed in the cytoplasm of *E. coli*. With the established in silico screening method, some of the putative DmoA enzymes were identified, characterized, and made available for the scientific community. The novel finding of DmoA’s oxidoreductase activity suggests that it could serve as an oxidoreductase biocatalyst for industrial applications such as cofactor regeneration systems. The identified four soluble DMS monooxygenases could be coupled with the potential DmoB for the bioremediation process.

## Supplementary Information

Below is the link to the electronic supplementary material.

Supplementary file1 (PDF 760 kb)

## Data Availability

The datasets generated during and/or analyzed during the current study are available from the corresponding author on reasonable request.
