# Supplementary material for: In silico screening and heterologous expression of soluble dimethyl sulfide monooxygenases of microbial origin in Escherichia coli
Source: Appl Microbiol Biotechnol. 2022 Jun 17;106(12):4523–37. doi: 10.1007/s00253-022-12008-8 (PMC9259527; doi:10.1007/s00253-022-12008-8)
Supplement: Supplementary file 1 — Supplementary file1 (PDF 760 kb) [file 253_2022_12008_MOESM1_ESM.pdf]

In silico screening and heterologous expression of soluble dimethyl sulfide monooxygenases of microbial origin in *Escherichia coli*

Prasanth Karaiyan [1], Catherine Ching Han Chang[1,2], Chan Eng-Seng[1], Tey Beng Ti[1,3], Ramakrishnan Nagasundara Ramanan [1,2*], Ooi Chien Wei [1,3*]

[1] Chemical Engineering Discipline, School of Engineering, Monash University Malaysia, Jalan Lagoon Selatan, 47500 Bandar Sunway, Selangor, Malaysia.

[2] Arkema Thiochemicals Sdn. Bhd., Jalan PJU 1A/7A OASIS Ara Damansara, 47301 Petaling Jaya, Selangor Darul Ehsan, Malaysia.

[3] Advanced Engineering Platform, Monash University Malaysia, Jalan Lagoon Selatan, 47500, Bandar Sunway, Selangor, Malaysia.

Corresponding authors:

Dr. Ramakrishnan Nagasundara Ramanan (ramanan@monash.edu)

A/Prof. Ooi Chien Wei (ooi.chien.wei@monash.edu)

Supporting Information

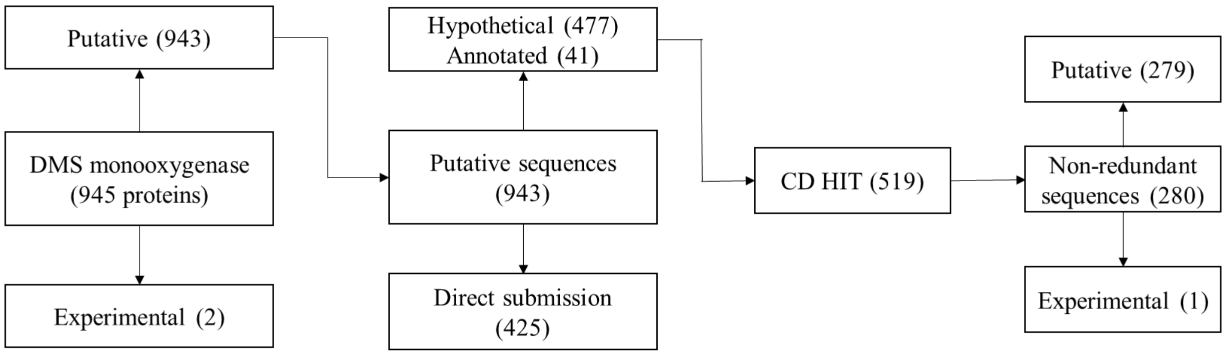

Figure S1: Screening of non-redundant putative sequences. Based on gene bank information, the putative sequences were classified as hypothetical, annotated and direct submission sequences. The genes encoding for alkanesulfonate monooxygenase and the direct submission sequences (425 proteins) were eliminated from the list because information on the method of annotation and whole genome sequencing were not available. Sequences with 100% identity but different gene accession IDs were grouped using CD-HIT as a single sequence to eliminate redundant sequences.

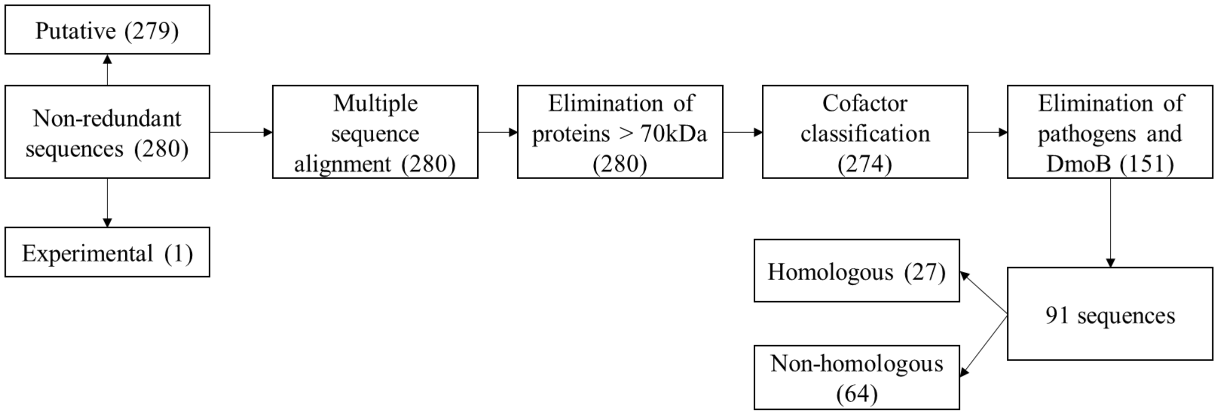

Figure S2: Classification of DMS monooxygenases. The size of the putative proteins varied from 11 to 109 kDa. Proteins with molecular weight less than 25 kDa could encode for DmoB, while proteins with molecular weight above 70 kDa could encode for fusion protein. Hence, proteins with molecular weight in the range of 40-60 kDa were selected. This screening exercise eliminated DmoB and fusion proteins (DmoA and DmoB). The expression of proteins with molecular weight above 60 kDa in *E. coli* are also challenging; hence, proteins with molecular weight higher than 60 kDa were eliminated from both homologous and non-homologous sets. Proteins with cofactors NADH and FMN were selected. Next, the proteins from human pathogenic origins were eliminated to remove the possibility of potential toxic proteins from the selection set.

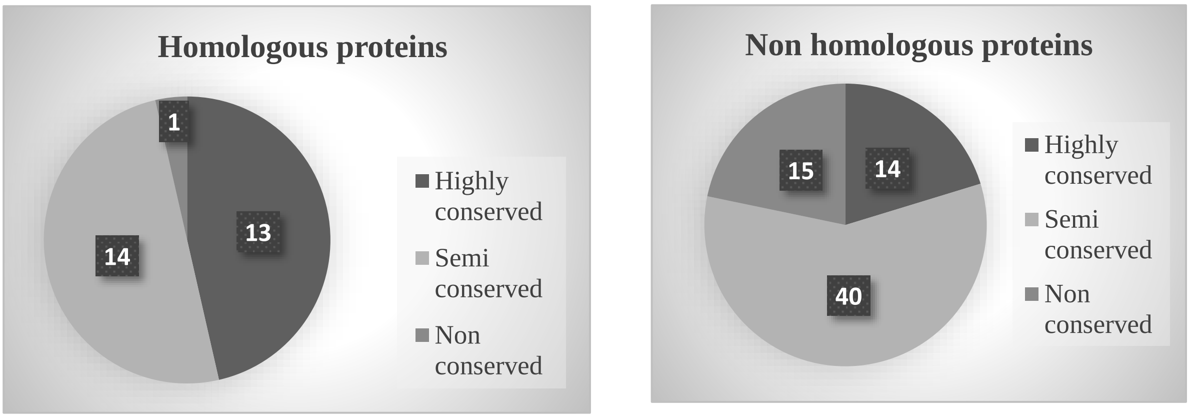

Figure S3: Conversation analysis of homologous and non-homologous. Sequences from homologous and non-homologous sets were subjected to conversation analysis using 'ClustalW' to classify them into 'highly conserved', 'semi-conserved', and 'non-conserved', based on 90%, 50% and < 20% identity with query sequence. Non-conserved proteins were removed because several amino acid substitutions were seen in the active and binding sites. The category with the highest number of putative sequences from both sets were subjected to protein solubility prediction.

Table S1: Selected DMS monooxygenases

Gene accession ID	Gene size (bp)	Classification [A]	Binding residues [B]		
			N-terminal	Middle region	C-terminal
E9JFX9.1	1461	Highly conserved (homologous)	FMHQ, ADVYQ, SYY	AASGF	GLG
KIR16147.1	1437	Highly conserved (homologous)	FMHQ, ADVYQ, SYY	AAS(G-S)F	G(L-F)G
AKP76571.1	1412	Semi conserved (homologous)	FMH(Q-Y), ADVYQ, SYY	AAS(G-K)F	GLG
ODA67029.1	1290	Semi conserved (homologous)	FMHQ, ADV- No match	AAS(G-P)F	GLG
PMQ09592.1	1410	Semi conserved (non-homologous)	FMHQ, ADVYQ, (S-G)YY	A(A-T)S(G-P)F	G(L-R)G
KXZ64626.1	1431	Semi conserved (non-homologous)	FMH(Q-I), AD(V-I)YQ, (S-G)YY	AAS(G-E)F	G(L-R)(G-Y)
KPC99150.1	1200	Semi conserved (non-homologous)	FMH(Q-N), ADVYQ, SYY	A(A-S)S(G-E)F	(G-T)(L-R)(G-E)

[A]: Classification based on conserved residues % identity with query sequence.

[B]: Conserved binding residues with its position F10, M12, H17, Q18, ADV(57-59) Y63, Q79, S137, Y138, Y158, A 227, ASG(229-231), F245, GLG(366-368) and the amino acid substitutions with respect to E9FX9.1 were given in parenthesis.

Table S2: MFE values for Gensmart-and COOL-optimized sequences

**Gene**	**Total MFE**		**5'TSS**		**RBS**		**Start codon**	
	**Gensmart (kcal/mol)**	**COOL (kcal/mol)**	**Gensmart (kcal/mol)**	**COOL (kcal/mol)**	**Gensmart (kcal/mol)**	**COOL (kcal/mol)**	**Gensmart (kcal/mol)**	**COOL (kcal/mol)**
dmoA1	-27.7	-29.1	-19.4	-18	-3.5	-2.1	-0.6	-3.7
dmoA3	-29	-26.2	-18.9	-18.6	-3	-2.7	-2.5	-2.3
dmoA4	-34.4	-25.1	-15.9	-15.9	0	0	-4.5	-0.6
Others (*dmoA2*, *A5*,*A6*,*A7*)	-25.9	-25.9	-15.9	-15.9	0	0	Open	Open

Note: SUMO tag was added into the gene sequence of *dmoA2*, *dmoA5*, *dmoA6* and *dmoA7*. Hence, MFE values calculated by both tools for these genes were similar. However, Gensmart-optimized sequences were found to have a higher codon adaptation index values than COOL-optimized sequences. From the MFE values and mRNA secondary structure diagram (data not shown), the sequences with an open conformation of RBS, a start codon and a MFE value higher than -6 kcal/mol were chosen. Though the total MFE values for all the sequences were highly negative, the MFE of RBS, and start codon were higher than -6 kcal/ mol.

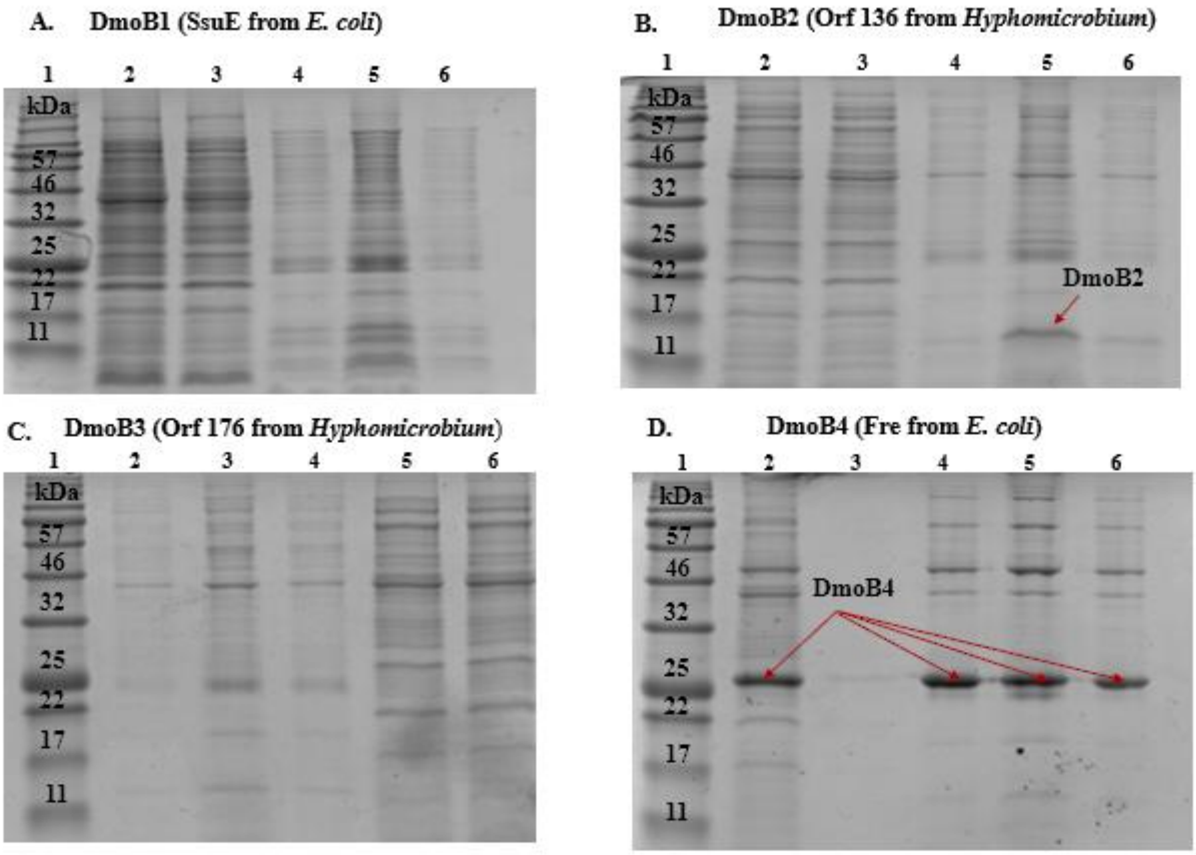

Figure S4: SDS-PAGE analysis of His-tag purified DmoB. A and B: Lane 1: Protein marker, lane 2: load, lane 3: Flow through, lane 4: Wash, lane 5: Elution 1, lane 6: Elution 2. C: Lane 1: Protein marker, lane 2: Elution 1, lane 3: Elution 2, lane 4: Wash, lane 5: Flow through, lane 6: load. D: Lane 1: Protein marker, lane 2: load, lane 3: Wash, lane 4: Elution 1, lane 5: Elution 2, lane 6: Elution 3.

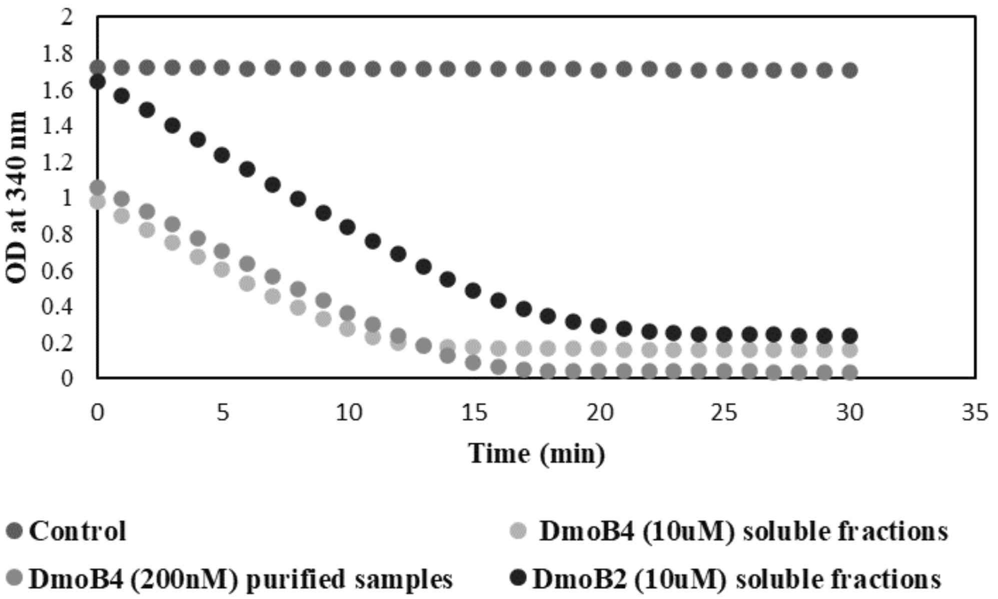

Figure S5: NADH:FMN oxidoreduction activity for DmoB in the presence of 3µM FMN.

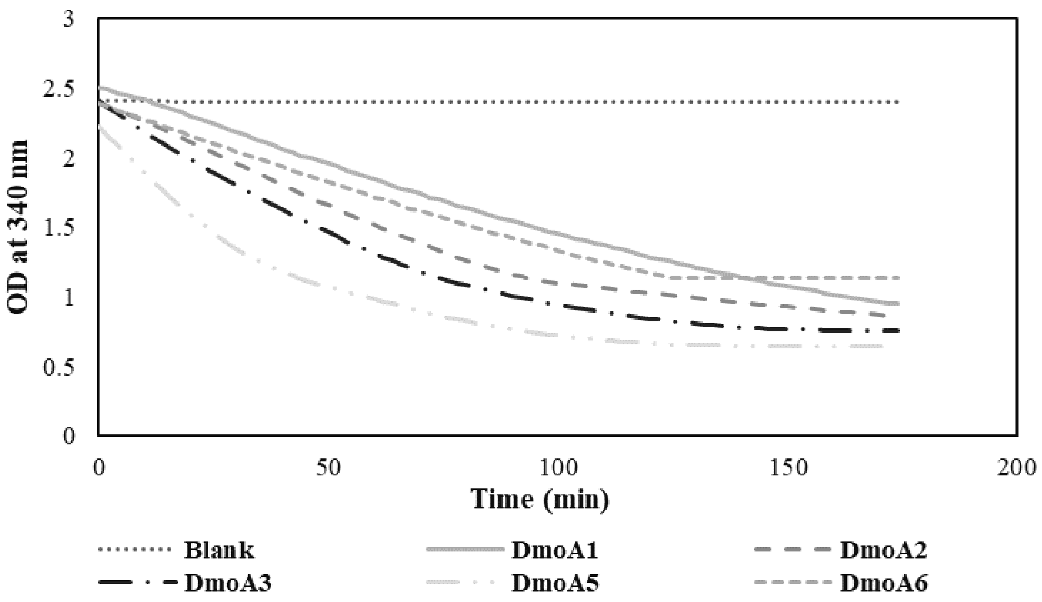

Figure S6: NADH oxidation activity for DmoA in the absence of FMN. Control: Cell-free extracts from [pET-28-b (+)] *E. coli* BL21 (DE3) star cells.

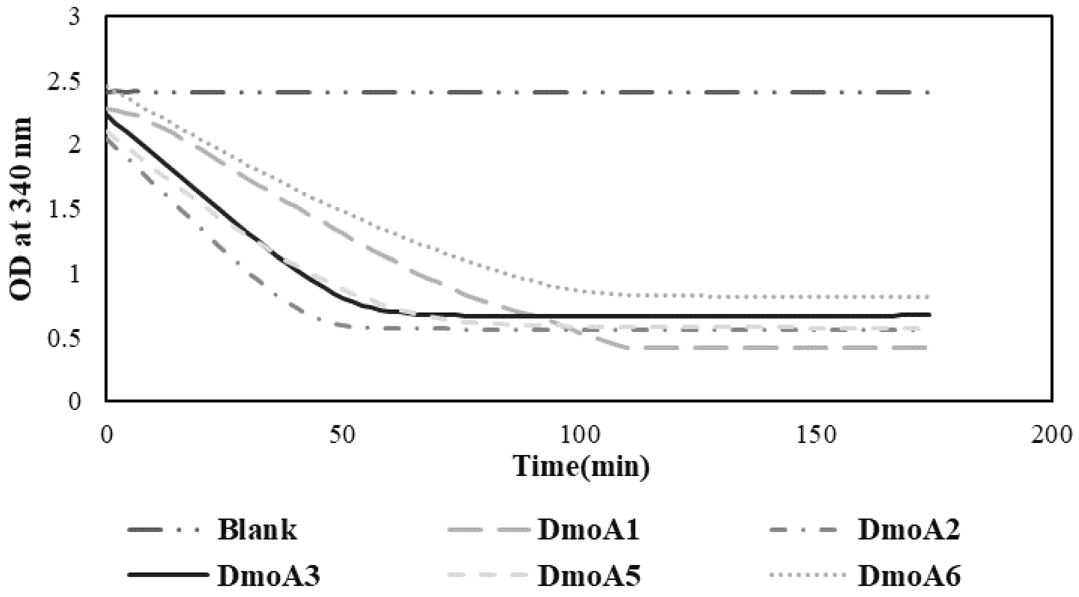

Figure S7: NADH:FMN oxidoreduction activity for DmoA in the presence of 3µM FMN. Control: Cell-free extracts from [pET-28-b (+)] *E. coli* BL21 (DE3) star cells.

Table S3: Biocatalytic reaction conditions for DmoAB

Protein	Fractions	DmoA and DmoB loading (%)	DMS (mM)	NADH (mM)	FMN (μM)	DTT (μM)	Mohr's salt (μM)	Buffers	Time interval	MT production (Yes/No)
DmoA1	Soluble and His-tag purified samples	10,20, 40, 60 and 80	1,5, and 10	1	3	5	5	20mM PIPES-HCl, pH: 7.4, 1× PBS, buffer pH: 7.4, 50mM phosphate buffer, pH: 7.2	10 min, 15 min, 30 min, 1 hr, 3 hrs, 6 hrs, 14 hrs.	No
DmoA2										No
DmoA3										No
DmoA4										No
DmoA5										No
DmoA6										No
DmoA7										No
DmoAB (2.5 mg/mL)										No
Negative control	Nil	Nil								No
